# *VNN1* Gene Expression Levels and the G-137T Polymorphism Are Associated with HDL-C Levels in Mexican Prepubertal Children

**DOI:** 10.1371/journal.pone.0049818

**Published:** 2012-11-21

**Authors:** Leonor Jacobo-Albavera, Pablo I. Aguayo-de la Rosa, Teresa Villarreal-Molina, Hugo Villamil-Ramírez, Paola León-Mimila, Sandra Romero-Hidalgo, Blanca E. López-Contreras, Fausto Sánchez-Muñoz, Rafael Bojalil, Juan Antonio González-Barrios, Carlos A. Aguilar-Salinas, Samuel Canizales-Quinteros

**Affiliations:** 1 Unidad de Biología Molecular y Medicina Genómica, Instituto Nacional de Ciencias Médicas y Nutrición “Salvador Zubirán” (INCMNSZ), Mexico City, Mexico; 2 Departamento de Biología, Facultad de Química, Universidad Nacional Autónoma de México (UNAM), Mexico City, Mexico; 3 Instituto Nacional de Medicina Genómica (INMEGEN), Mexico City, Mexico; 4 Departamento de Inmunología, Instituto Nacional de Cardiología “Ignacio Chávez”, Mexico City, Mexico; 5 Departamento de Cuidado de la Salud, Universidad Autónoma Metropolitana (UAM) Xochimilco, Mexico City, Mexico; 6 Laboratorio de Medicina Genómica, Hospital Regional “Primero de Octubre”, Instituto de Seguridad y Servicios Sociales de los Trabajadores del Estado (ISSSTE), Mexico City, Mexico; 7 Departamento de Endocrinología y Metabolismo, INCMNSZ, Mexico City, Mexico; Ohio State University Medical Center, United States of America

## Abstract

**Background:**

*VNN1* gene expression levels and the G-137T polymorphism have been associated with high density lipoprotein cholesterol (HDL-C) levels in Mexican American adults. We aim to evaluate the contribution of *VNN1* gene expression and the G-137T variant to HDL-C levels and other metabolic traits in Mexican prepubertal children.

**Methodology/Principal Findings:**

*VNN1* mRNA expression levels were quantified in peripheral blood leukocytes from 224 unrelated Mexican-Mestizo children aged 6–8 years (107 boys and 117 girls) and were genotyped for the G-137T variant (rs4897612). To account for population stratification, a panel of 10 ancestry informative markers was analyzed. After adjustment for admixture, the TT genotype was significantly associated with lower *VNN1* mRNA expression levels (*P* = 2.9 × 10^−5^), decreased HDL-C levels (β = −6.19, *P* = 0.028) and with higher body mass index (BMI) z-score (β = 0.48, *P* = 0.024) in the total sample. In addition, *VNN1* expression showed a positive correlation with HDL-C levels (r = 0.220; *P* = 0.017) and a negative correlation with BMI z-score (r = −0.225; *P* = 0.015) only in girls.

**Conclusion/Significance:**

Our data suggest that *VNN1* gene expression and the G-137T variant are associated with HDL-C levels in Mexican children, particularly in prepubertal girls.

## Introduction

Low plasma levels of high-density lipoprotein cholesterol (HDL-C) is the most common dyslipidemia in Mexican children and adults [1]–[3], which is modulated by both genetic and lifestyle factors [4], [5]. Several genetic variants affecting HDL-C levels have been identified through candidate gene/genome-wide association studies (GWAS) and replicated in different populations [6]–[8]. However, relatively few genome-wide quantitative gene expression studies identifying expression quantitative trait loci (eQTLs) for HDL-C levels have been reported [9]–[12]. Interestingly, these studies have identified some genes associated with HDL-C levels not previously associated with this trait by GWAS. In this regard, Göring et al. [9] reported that Vanin 1 (*VNN1*) gene expression levels showed the strongest correlation with HDL-C concentrations in lymphocytes of Mexican-American adults. Moreover, the authors also observed that the functional G-137T polymorphism (rs4897612) was associated with both *VNN1* expression levels and HDL-C concentrations in this population, even though the polymorphism had not been previously associated with HDL-C levels in GWAS performed mainly in Caucasians [6]–[8]. Although there is no direct evidence of the role of the *VNN1* protein in HDL-C levels regulation, it is known that *VNN1* codes for pantetheinase and produces cysteamine, a potent antioxidant that prevents lipid peroxidation [13], [14] and has been associated with other metabolic traits such as obesity in animal models [15]. *VNN1* thus can be considered as a reasonable candidate gene to modulate HDL-C levels and perhaps other metabolic traits.

In addition to genetic variation, it is known that environmental factors may modify gene expression [16], [17]. Because environmental exposure is likely to be not as relevant in children than in adults [18], [19], we sought to replicate the *VNN1* expression association with the G-137T variant and HDL-C levels in a population sample of Mexican prepubertal children, and to assess whether *VNN1* expression is also associated with other metabolic traits.

## Materials and Methods

### Subjects

We analyzed 224 healthy unrelated school-aged Mexican-Mestizo children (107 boys and 117 girls) aged 6 to 8 years, recruited from a summer camp for children of employees of the Mexican Health Ministry (Convivencia Infantil 2008 and 2009, Secretaría de Salud). Weight, height and waist circumference were measured in all participants. Body mass index (BMI) was calculated as weight in kilograms divided by height in meters squared. BMI z-scores were calculated using age and sex specific BMI reference data, as recommended by the Centers for Disease Control and Prevention [20]. Fat mass percentage was measured using a bioelectric impedance method (Quantum × impedance analyzer, RJL Systems, Detroit, MI). None of the participants had evidence of diabetes, thyroid, renal or liver disease. A parent of each child signed the consent form for participation. The project was approved by the Institutional Committee of Biomedical Research in Humans of the Instituto Nacional de Ciencias Médicas y Nutrición Salvador Zubirán (INCMNSZ).

**Table 1:** Anthropometric and biochemical parameters according to gender.

Characteristic	Total subjects (224)	Boys (107)	Girls (117)	*P* a
Age, years	7.73±0.97	7.84±0.97	7.63±0.96	0.194
BMI z-score	0.99±0.98	1.0±1.0	0.98±0.97	0.846
FM, %	30.03±10.52	28.37±11.60	31.52±9.25	0.041
TG, mg/dL	98.03±59.09	90.95±50.38	104.50±65.62	0.087
TC, mg/dL	175.83±31.39	173.36±29.90	178.09±32.66	0.261
HDL-C, mg/dL	48.58±10.56	49.15±10.36	48.06±10.75	0.442
ApoA1, mg/dL	168.52±21.74	171.08±20.57	166.40±22.53	0.120
HA (%)	19.6	16.8	22.2	0.310^b^

Data are means ± s.d. or n (%). BMI, body mass index; FM, percent fat mass; TG, triglyceride; TC, total cholesterol; HDL-C, high-density lipoprotein cholesterol; HA, hypoalphalipoproteinemia.

a *P*-values were calculated by t-test;

b X^2^ test.

### Biochemical Parameters

Blood samples were drawn from all participants after a 12-hour fast. Total cholesterol (TC), triglyceride (TG), HDL-C and ApoA1 plasma levels were measured at the INCMNSZ with commercially available standardized methods as described by Villarreal-Molina et al. [21]. Hypoalphalipoproteinemia (HA) was defined as HDL-C levels<40 mg/dL [22].

**Table 2:** Correlation of *VNN1* mRNA levels in leukocytes with metabolic phenotypes.

	Whole Population (n = 224)			Boys (n = 107)			Girls (n = 117)		
Metabolic phenotypes	r	*P*	*P* a	r	*P*	*P* a	r	*P*	*P* a
BMI z-score	−0.144	0.032	0.034	−0.062	0.528	0.518	−0.225	0.015	0.015*
FM, %	−0.118	0.080	0.085	−0.068	0.490	0.525	−0.145	0.118	0.119
TG, mg/dL	−0.149	0.026	0.028	−0.062	0.525	0.576	−0.208	0.025	0.023
TC, mg/dL	−0.056	0.402	0.396	−0.047	0.631	0.643	−0.051	0.586	0.586
HDL-C, mg/dL	0.086	0.198	0.211	−0.071	0.465	0.448	0.220	0.017	0.017
ApoA1, mg/dL	0.042	0.546	0.566	−0.106	0.308	0.308	0.129	0.170	0.175

BMI, body mass index; FM, percent fat mass; TG, triglyceride; TC, total cholesterol; HDL-C, high-density lipoprotein cholesterol.

a *P*-values adjusted for admixture.

* Significant after Bonferroni correction.

### *VNN1* mRNA Expression Analysis

Total RNA was extracted from leukocytes using TRIzol (Invitrogen, Life Technologies, Carlsbad, CA, USA). To avoid DNA carryover, DNAse treatment was performed with DNAse I recombinant (Roche, Rotkreuz, Switzerland). To perform the expression analysis, 1000 ng of total RNA was reverse transcribed with TaqMan Reverse Transcription Reagents (Applied Biosystems, Foster City, CA) using random hexamers, according to the protocol recommended by the manufacturer. Real-time PCR was performed in a LightCycler 2.0 (Roche, Rotkreuz, Switzerland), using LNA TaqMan probes from the Universal Probe Library (Roche, Rotkreuz, Switzerland), in combination with intron-spanning specific primers as described previously [23]. The following primers and probes were used to assess *VNN1* gene expression in human leucocytes: tcctgaggtgttgctgagtg (forward), agcgtccgtcagttgacac (reverse), and probe #80 (cat. no. 04689038001). Hipoxanthine phosphoribosyl transferase (*HPRT*) expression was measured as reference using *HPRT* primers tgatagatccattcctatgactgtaga (forward), caagacattctttccagttaaagttg (reverse), and probe #22 (cat. no. 04688961001); *β-actin* (*ACTB*) was measured as reference using Universal ProbeLibrary Human *ACTB* Gene Assay (cat. no. 05046165001). All assays showed linearity and a coefficient of variation<10%. Relative quantification of gene expression was calculated with the LightCycler Software 4.0.

**Figure 1:**
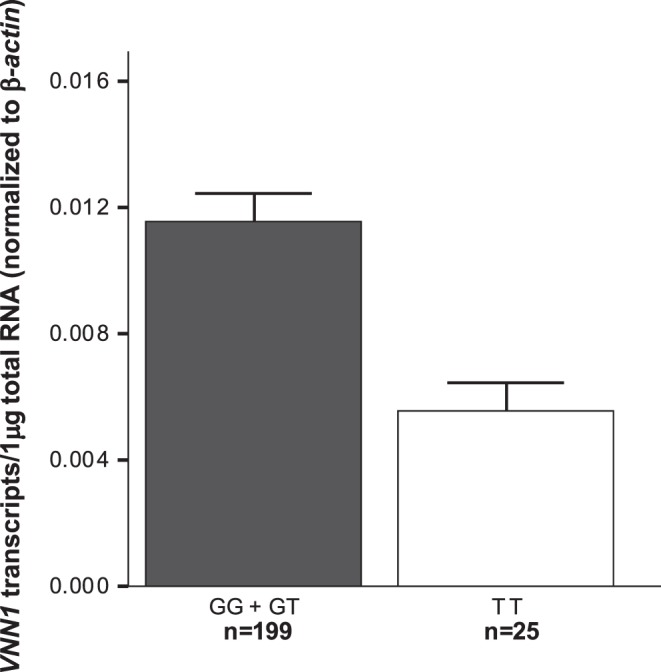
Effect of G-137T variant on *VNN1* expression levels in Mexican prepubertal children. The results are presented as the global mean ± the standard error.

**Table 3:** Association of G-137T variant with metabolic parameters stratified by gender.

	All children (n = 224)			Boys (n = 107)			Girls (n = 117)		
Parameters	Effect (SE)	*P*	*P* a	Effect (SE)	*P*	*P* a	Effect (SE)	*P*	*P* a
BMI z-score	0.48 (0.21)	0.024	0.024	0.69 (0.36)	0.061	0.063	0.38 (0.26)	0.146	0.156
FM, %	5.66 (2.25)	0.012	0.012*	6.45 (4.24)	0.131	0.123	4.56 (2.46)	0.067	0.072
TG, mg/dL	25.76 (12.45)	0.040	0.194	−1.65 (17.63)	0.926	0.371	39.53 (17.34)	0.024	0.079
TC, mg/dL	16.35 (6.58)	0.014	0.034	10.64 (10.41)	0.309	0.423	18.93 (8.64)	0.031	0.057
HDL-C, mg/dL	−6.19 (2.20)	0.005	0.028	−2.10 (3.62)	0.562	0.994	−8.47 (2.80)	0.003	0.010*
ApoA1 mg/dL	−9.66 (4.77)	0.044	0.093	−5.95 (8.72)	0.497	0.969	−12.44 (5.98)	0.040	0.093

Effect values are presented as effect for two T copies (recessive model), standard error (SE). Genotype frequencies: all children (GG, 41.1%; GT, 47.7%; TT, 11.2%); boys (GG, 42.1%; GT, 49.5%; TT, 8.4%); girls (GG, 40.2%; GT, 46.1%; TT, 13.7%). BMI, body mass index; FM, percent fat mass; TG, triglyceride; TC, total cholesterol; HDL-C, high-density lipoprotein cholesterol.

a *P*-values adjusted for admixture in all tests and for BMI z-score when appropriate.

* Significant after Bonferroni correction.

### Single Nucleotide Polymorphisms Genotyping

Genomic DNA was extracted from peripheral blood leukocytes using a QIAamp DNA Blood Kit (Qiagen, Valencia, California). The *VNN1* G-137T variant was genotyped using the TaqMan assay C_29857881_10 (ABI Prism 7900HT Sequence Detection System; Applied Biosystems, Foster City, CA). No discordant genotypes were observed in 30 duplicate samples analyzed by direct sequencing. *VNN1* G-137T genotype frequencies were tested for Hardy-Weinberg equilibrium, deviation was not observed in any group.

Because the Mexican population resulted from the admixture of mainly European (Spaniard) and Native American populations, it was necessary to assess whether any association could be confounded by population stratification. A panel of 10 ancestry informative markers (AIMs) distinguishing mainly Native American and European ancestry (δ for the minor allele>0.29), was screened in all participants (rs3340, rs1881826, rs2341823, rs4130405, rs1980888, rs1487214, rs726391, rs724729, rs292932 and rs1877751) [24], [25]. Genotyping was performed using TaqMan assays (ABI Prism 7900HT Sequence Detection System; Applied Biosystems, Foster City, CA). Genotyping call rates of each ancestry informative marker exceeded 95%, and no discordant genotypes were observed in 48 duplicate samples. No ancestry informative marker showed significant departure from Hardy-Weinberg equilibrium.

### Statistical Analysis

Because gene expression, HDL-C levels and the effect of some polymorphisms on metabolic traits are known to have gender differences [26]–[29], all analyses were performed on the entire sample and stratified by gender. Differences in anthropometric and biochemical parameters were analyzed using Student’s t-test. Because *VNN1* gene expression and triglyceride levels were not normally distributed, they were log transformed for analysis. Correlations between *VNN1* gene expression and metabolic parameters were analyzed using Pearson’s correlation and partial correlation was used to examine the correlations between gene expression and metabolic phenotypes adjusting for admixture. Differences in anthropometric and biochemical parameters according to G-137T genotype were tested by linear regression analysis. Associations were analyzed using additive (Table S1), dominant (Table S2) and recessive models for the T allele, being the recessive model the most significant. Comparison of *VNN1* expression levels between G-137T genotypes was performed using Mann-Whitney U test. All statistical analyses were performed using SPSS version 15.0 (Chicago, IL) and a *P*-value of less than 0.05 was considered statistically significant. Genetic ancestry estimates were computed from genotypes for 10 AIMs using the program ADMIXMAP [30]. Because the Mexican-Mestizo population derived mainly from Native American and European populations, the model used included two primary parental populations. All analyses were adjusted for admixture and for BMI-z score when appropriate. To address multiple testing, we first determined the average pairwise correlation for all measurements (6 measurements, correlation = 0.38); with this correlation we calculated Bonferroni’s correction using the freely available Simple Interactive Statistical Analyses Software (http://www.quantitativeskills.com/sisa/) and a *P*-value below 0.017 was considered significant. The study power to detect association of G-137T with HDL-C levels was estimated using QUANTO software (v1.2.4, http://hydra.usc.edu/GxE/) and reached 82.8%, assuming a recessive model with a minor allele (T) frequency of 0.35, beta for HDL-C levels of −6.19 (SE = 2.20) and sample size of 224 individuals. Statistical power stratified by gender was 53.5% for boys and 54.1% for girls.

## Results

### Anthropometric and Biochemical Characteristics

Table 1 describes the anthropometric characteristics and lipid profile of the children stratified by gender. Fat mass percentage was significantly higher in girls (*P* = 0.041), but no significant gender differences in BMI-z score or lipid parameters were observed.

### *VNN1* Expression Analysis

*VNN1* expression levels were not significantly correlated with HDL-C levels in the entire study population, but showed a significant negative correlation with BMI z-score and TG levels (*P* = 0.032 and 0.026, respectively) (Table 2). On stratification according to gender, a significant positive correlation of *VNN1* expression levels with HDL-C (*P* = 0.017), and significant negative correlations with BMI z-score and TG levels were observed exclusively in girls (*P* = 0.015 and *P* = 0.025). All associations remained significant by further adjustment for admixture, but only the association with BMI-z score was significant after correction for multiple testing.

### G137T Variant, *VNN1* Expression Levels and Metabolic Parameters

The overall frequency of the T-137 allele was 35%. The TT genotype was significantly associated with lower *VNN1* expression levels (*P* = 2.9 × 10^−5^ after adjusting for admixture) (Figure 1). Multiple linear regression analyses to predict *VNN1* expression levels revealed that only TT genotype contributed independently to explain 4.8% of the variance at *VNN1* gene expression (*P* = 0.004). In addition, the TT genotype was associated with lower HDL-C and ApoA1 levels (*P* = 0.005 and 0.044, respectively), and with higher BMI z-score, percent fat mass, TG and TC levels in the whole sample (*P*<0.05, Table 3). After adjusting for BMI z-score and admixture, only associations with TC and HDL-C remained significant in the whole sample (*P* = 0.034 and 0.028, respectively). On stratification according to gender, a significant association of TT genotype with lower HDL-C levels was observed exclusively in girls (*P* = 0.010).

## Discussion

Several epidemiological studies report that the onset of metabolic disease is occurring at increasingly earlier ages [31], [32]. In the Mexican population, low HDL-C levels is the most common dyslipidemia in both adults and adolescents [1]–[3], and was also found to be common in prepubertal children aged 6–8 years (20%), showing that low HDL-C levels are present since childhood in this population.

Several studies have shown that variation in gene expression is probably an important mechanism influencing HDL-C levels [9]–[11], [33]. Göring et al. observed that *VNN1* expression levels showed the strongest correlation with HDL-C concentrations in lymphocytes of Mexican-American adults, (r = 0.28, *P* = 4 × 10^−9^). Similarly, we observed a positive correlation of *VNN1* expression levels in leukocytes with HDL-C levels in Mexican prepubertal children, which was significant only in girls (r = 0.22, *P* = 0.017). We also observed a negative correlation of *VNN1* expression levels with triglyceride levels and BMI z-score in the whole sample, again with gender-specific differences. Although the mechanism by which *VNN1* function may affect these metabolic traits is largely unknown, there is experimental evidence that Vanin 1 is a central regulator of lipid biosynthesis by controlling the flux through the fatty acid and/or cholesterol biosynthetic pathways [34]. Because to our knowledge this is the only study analyzing the effects of *VNN1* expression levels on metabolic traits in children, further studies are required to confirm this finding and to further characterize the gender differences observed.

The *VNN1* T-137 allele frequency in the Mexican children population (35%) was similar to the 33% frequency reported in Mexican-American population and lower than in European populations (67%) [35]. Several studies have reported an effect of genetic ancestry on both global gene expression and associations of gene variants with disease [36], [37]. Because the Mexican population is the result of admixture, mainly of Spanish and Native American populations [25], [38], we included ancestry as a confounding factor to test for associations. Although the panel of AIMs analyzed is small (10 AIMs), the mean admixture proportions of the population were estimated as 65% Native American and 35% European, in agreement with previous studies in the population of Mexico City using 69 AIMs [25]. All associations reported in the present study remained significant after adjusting for admixture, suggesting that population stratification was not an important confounding factor in our analysis.

The TT genotype was significantly associated with lower *VNN1* expression and HDL-C levels, in accordance with the findings of Göring et al. in Mexican-American adults. Although experimental evidence strongly supports that this promoter polymorphism has functional consequences with respect to binding transcription factors [9], this genotype contributed independently to explain only 4.8% of the variance at *VNN1* gene expression. While this is consistent with the previously reported range of the effect of SNPs on gene expression variability [11], the genetic and environmental factors explaining most of *VNN1* gene expression variability remain to be determined.

Interestingly, associations of the TT genotype with higher TG and TC levels were observed in girls. However, these associations lost significance after adjusting for BMI z-score, suggesting they may be mediated by an effect on body fat. To our knowledge, there is no previous experimental evidence of the role of *VNN1* in human adipocyte function, however it was identified as an obesity-related gene in mice [15] and is known to induce lipolysis in rat adipose tissue [39]. Because *VNN1* gene variants have not been previously found to be associated with obesity in GWAS [40], [41] and because the sample number analyzed here is reduced, these associations should be interpreted with caution. Additional studies are required to confirm and further understand the role of *VNN1* in human obesity.

The gender differences observed are noteworthy, particularly because it involved prepubertal children. Mexican prepubertal girls showed significantly higher fat mass percentage than boys, which could explain at least part of the gender differences observed for genetic associations and correlations. Gender differences in the effect of polymorphisms on lipid traits have been previously observed in prepubertal children [28], apparently explained by dehydroepiandrostenone-sulfate (DHEA-S) level variation [42]. Because we did not measure DHEA-S levels, we cannot rule out whether this factor modulates the effect of *VNN1* gene expression levels and the G-137T variant on metabolic traits in this study.

In conclusion, our data suggest that *VNN1* gene expression levels and the G-137T variant are associated with lipid traits (particularly HDL-C levels) in Mexican prepubertal girls. However, these results should be interpreted with caution and further studies with larger samples sizes are required to confirm these findings.

## Supporting Information

Table S1 **Association of G-137T variant with metabolic parameters stratified by gender (additive model).**
(DOC)

Table S2 **Association of G-137T variant with metabolic parameters stratified by gender (dominant model).**
(DOC)
