# Supplementary material for: VNN1 Gene Expression Levels and the G-137T Polymorphism Are Associated with HDL-C Levels in Mexican Prepubertal Children
Source: PLoS One. 2012 Nov 21;7(11):e49818. doi: 10.1371/journal.pone.0049818 (PMC3504107; doi:10.1371/journal.pone.0049818)
Supplement: Table S1 — Association of G-137T variant with metabolic parameters stratified by gender (additive model). (DOC) [file pone.0049818.s001.doc]

**Table S1**. Association of G-137T variant with metabolic parameters stratified by gender (additive model)

|  | All children (n=224) | | | Boys (n=107) | | | Girls (n=117) | | |
| --- | --- | --- | --- | --- | --- | --- | --- | --- | --- |
| Parameters | Effect (SE) | *P* | *P^a^* | *Effect (SE)* | *P* | *P^a^* | *Effect (SE)* | *P* | *P^a^* |
| BMI *z-*score | 0.14 (0.10) | 0.174 | 0.185 | 0.16 (0.16) | 0.321 | 0.304 | 0.13 (0.13) | 0.342 | 0.335 |
| FM, % | 2.07 (1.07) | 0.054 | 0.060 | 2.16 (1.83) | 0.242 | 0.278 | 1.75 (1.24) | 0.162 | 0.158 |
| TG, mg/dL | 6.38 (6.00) | 0.289 | 0.600 | -1.66 (7.82) | 0.832 | 0.479 | 11.58 (8.84) | 0.193 | 0.307 |
| TC, mg/dL | 7.80 (3.15) | 0.014 | 0.024 | 2.06 (4.64) | 0.658 | 0.807 | 11.88 (4.29) | 0.007 | 0.011* |
| HDL-C, mg/dL | -1.90 (1.07) | 0.077 | 0.164 | -0.06 (1.61) | 0.969 | 0.758 | -3.23 (1.43) | 0.025 | 0.040 |
| ApoA1 mg/dL | -3.70 (2.26) | 0.103 | 0.158 | -1.90 (3.42) | 0.581 | 0.499 | -4.46 (3.03) | 0.144 | 0.247 |

Effect values are presented as effect for an additive model, standard error (SE). BMI, body mass index; FM, percent fat mass; TG, triglyceride; TC, total cholesterol; HDL-C, high-density lipoprotein cholesterol. ^a^*P*-values adjusted for admixture in all tests and for BMI z-score when appropriate. *Significant after Bonferroni correction.
