# Supplementary material for: VNN1 Gene Expression Levels and the G-137T Polymorphism Are Associated with HDL-C Levels in Mexican Prepubertal Children
Source: PLoS One. 2012 Nov 21;7(11):e49818. doi: 10.1371/journal.pone.0049818 (PMC3504107; doi:10.1371/journal.pone.0049818)
Supplement: Table S2 — Association of G-137T variant with metabolic parameters stratified by gender (dominant model). (DOC) [file pone.0049818.s002.doc]

**Table S2**. Association of G-137T variant with metabolic parameters stratified by gender (dominant model)

|  | All children (n=224) | | | Boys (n=107) | | | Girls (n=117) | | |
| --- | --- | --- | --- | --- | --- | --- | --- | --- | --- |
| Parameters | Effect (SE) | *P* | *P^a^* | *Effect (SE)* | *P* | *P^a^* | *Effect (SE)* | *P* | *P^a^* |
| BMI *z-*score | 0.05 (0.13) | 0.700 | 0.735 | 0.05 (0.20) | 0.811 | 0.777 | 0.06 (0.18) | 0.756 | 0.722 |
| FM, % | 1.41 (1.43) | 0.325 | 0.357 | 1.50 (2.29) | 0.515 | 0.599 | 1.17 (1.75) | 0.504 | 0.475 |
| TG, mg/dL | 0.86 (8.04) | 0.915 | 0.908 | -2.15 (9.91) | 0.829 | 0.684 | 3.14 (12.42) | 0.801 | 0.836 |
| TC, mg/dL | 7.25 (4.25) | 0.089 | 0.094 | -0.06 (5.88) | 0.992 | 0.901 | 13.85 (6.05) | 0.024 | 0.028 |
| HDL-C, mg/dL | -0.86 (1.44) | 0.550 | 0.637 | 0.57 (2.04) | 0.782 | 0.702 | -2.14 (2.03) | 0.293 | 0.281 |
| ApoA1 mg/dL | -2.75 (3.03) | 0.366 | 0.405 | -2.48 (4.26) | 0.561 | 0.418 | -2.60 (4.29) | 0.546 | 0.656 |

Effect values are presented as effect for a dominant model, standard error (SE). BMI, body mass index; FM, percent fat mass; TG, triglyceride; TC, total cholesterol; HDL-C, high-density lipoprotein cholesterol. ^a^*P*-values adjusted for admixture in all tests and for BMI z-score when appropriate.
